# Suppression of the antiferromagnetic pseudogap in the electron-doped high-temperature superconductor by protect annealing

**DOI:** 10.1038/ncomms10567

**Published:** 2016-02-04

**Authors:** M. Horio, T. Adachi, Y. Mori, A. Takahashi, T. Yoshida, H. Suzuki, L. C. C. Ambolode, K. Okazaki, K. Ono, H. Kumigashira, H. Anzai, M. Arita, H. Namatame, M. Taniguchi, D. Ootsuki, K. Sawada, M. Takahashi, T. Mizokawa, Y. Koike, A. Fujimori

**Affiliations:** 1 Department of Physics, University of Tokyo, Bunkyo-ku, Tokyo 113-0033, Japan; 2 Department of Engineering and Applied Sciences, Sophia University, Tokyo 102-8554, Japan; 3 Department of Applied Physics, Tohoku University, Sendai 980-8579, Japan; 4 KEK, Photon Factory, Tsukuba 305-0801, Japan; 5 Hiroshima Synchrotron Radiation Center, Hiroshima University, Higashi-Hiroshima 739-0046, Japan; 6 Graduate School of Science, Hiroshima University, Higashi-Hiroshima 739-8526, Japan; 7 Graduate School of Frontier Sciences, University of Tokyo, Kashiwa 277-0882, Japan

## Abstract

In the hole-doped cuprates, a small number of carriers suppresses antiferromagnetism and induces superconductivity. In the electron-doped cuprates, on the other hand, superconductivity appears only in a narrow window of high-doped Ce concentration after reduction annealing, and strong antiferromagnetic correlation persists in the superconducting phase. Recently, Pr_1.3−*x*_La_0.7_Ce_*x*_CuO_4_ (PLCCO) bulk single crystals annealed by a protect annealing method showed a high critical temperature of around 27 K for small Ce content down to 0.05. Here, by angle-resolved photoemission spectroscopy measurements of PLCCO crystals, we observed a sharp quasi-particle peak on the entire Fermi surface without signature of an antiferromagnetic pseudogap unlike all the previous work, indicating a dramatic reduction of antiferromagnetic correlation length and/or of magnetic moments. The superconducting state was found to extend over a wide electron concentration range. The present results fundamentally challenge the long-standing picture on the electronic structure in the electron-doped regime.

## Abstract (web summary)

In cuprates, superconductivity exists in a narrow window at high electron doping concentration with strong antiferromagnetic correlations. Here, the authors demonstrate superconductivity with no effect of antiferromagnetic order in a cuprate for a wide electron doping range following a protect anneal process.

## Graphical abstract

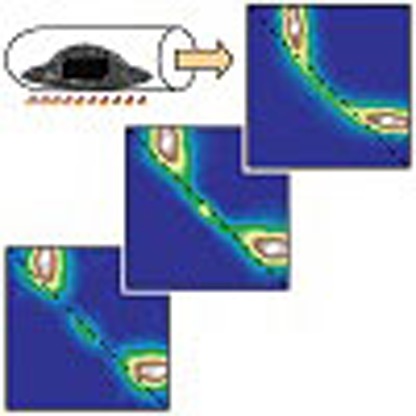

Since the discovery of the cuprate high-temperature superconductors, one of the central issues has been the relationship between antiferromagnetic (AFM) order or AFM spin fluctuations and superconductivity. Starting from the AFM parent insulator, a small amount (∼2%) of hole doping destroys the AFM ordering and superconductivity emerges. However, for the electron-doped high-temperature superconductors (e-HTSCs), the antiferromagnetism persists up to the optimum doping (∼15%). In the underdoped region of e-HTSCs, a large pseudogap opens due to AFM order or AFM correlation as observed by optical measurements^1^,^2^ and scanning tunneling spectroscopy^3^. Angle-resolved photoemission spectroscopy (ARPES) studies have shown that the pseudogap opens around the hot spots, namely, crossing points of the Fermi surface (FS) with the AFM Brillouin zone boundary in superconducting samples^4^,^5^,^6^. A neutron scattering study^7^ has revealed that the AFM correlation length is of order ∼10 lattice spacing in the superconducting phase, and the pseudogap in the ARPES spectra of the superconducting phase has been reproduced by assuming a similar AFM correlation length^8^,^9^.

Since the discovery of the e-HTSCs, it has been well known that annealing in a reducing atmosphere is necessary to realize superconductivity. As-grown samples are non-superconducting and AFM. By annealing the AFM-phase shrinks, and superconductivity appears^10^. Effects of annealing are still not fully understood on the microscopic level: a small amount of oxygen atoms at the apical oxygen site^11^,^12^, which stabilize the AFM ordering, and/or those at the regular site (in the CuO_2_ plane or the LnO (Ln: rare earth) layer)^13^ are possibly removed by reduction annealing, while the discovery of secondary phase of Ln_2_O_3_ created by annealing^14^ raised the possibility that annealing mends Cu defects existing in the as-grown sample by forming the Ln_2_O_3_ phase^15^,^16^. Previous ARPES studies have revealed that reduction annealing decreases the intensity of the AFM-folded bands and increase the spectral intensity at Fermi level (*E*_F_)^17^,^18^, but the AFM pseudogap has been seen in all the e-HTSCs from the underdoped to overdoped regions studied so far^19^. Therefore, the AFM pseudogap has been regarded as a hallmark of the e-HTSCs and the relationship between antiferromagnetism and superconductivity has been considered as a more essential ingredient of the e-HTSCs than the hole-doped ones.

In a previous study, Brinkmann *et al*.^20^ annealed thin single crystals of Pr_2−*x*_Ce_*x*_CuO_4_ sandwiched by Pr_2−*x*_Ce_*x*_CuO_4_ polycrystals of the same compositions and realized superconductivity with Ce concentration as low as 4%. Recently, in thin films^21^,^22^ and powdered samples^23^,^24^ of e-HTSCs, superconductivity was found even without Ce doping. Inspired by those studies, Adachi *et al*.^25^ further improved the reduction annealing method of Brinkmann *et al*. by using powders instead of polycrystals, and successfully synthesized bulk superconducting single crystals of Pr_1.3−*x*_La_0.7_Ce_*x*_CuO_4_ (PLCCO) with *x*=0.10. They call this new reduction annealing method protect annealing method. With protect annealing, one can protect the surface from over-reduction under a stronger reducing condition, that is, one can anneal the sample in a lower oxygen pressure for a longer time in a milder way, which leads to the smaller differences between bulk and surfaces and to the higher critical temperature (*T*_c_). Although PLCCO samples with such a low Ce concentration did not show superconductivity in previous studies^26^, the protect-annealed samples showed a *T*_c_ as high as 27.0 K (even higher than those prepared by conventional annealing, T. Adachi *et al*., unpublished.).

To study the effect of protect annealing on the electronic structure of PLCCO, we have performed ARPES measurements on single crystals of PLCCO with *x*=0.10 under different annealing conditions (Methods section). For sufficiently annealed samples, a sharp quasi-particle (QP) peak was observed on the entire FS without signature of an AFM pseudogap unlike all the previous studies, indicating dramatic reduction of AFM correlation length and/or of magnetic moments. By measuring the FS area, we also found that this superconducting state with suppressed antiferromagnetism was extended over a wide electron concentration range. The present study will call for a re-examination of the relationship between superconductivity and antiferromagnetism and of the phase diagram of the cuprate superconductors. Presented results for the annealed sample are those for annealed sample 1 with a *T*_c_ of 27.0 K out of three protect-annealed superconducting samples unless otherwise stated.

## Results

### Suppression of the AFM pseudogap

**Figure 1:**
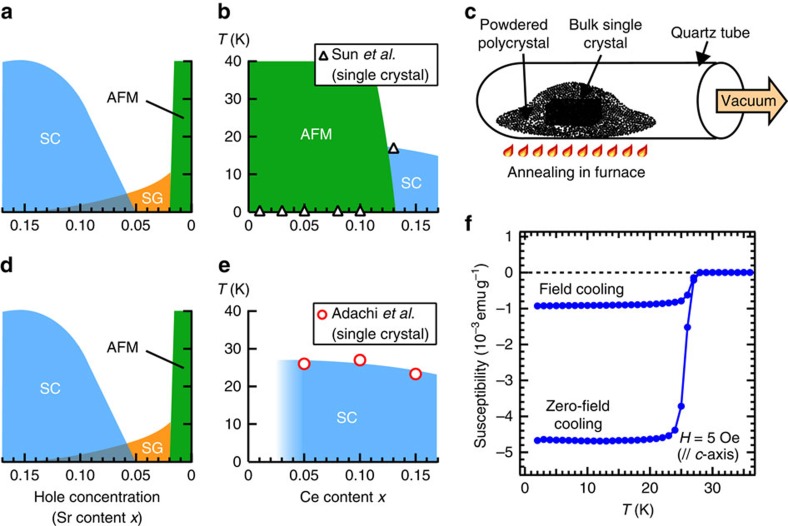
Superconducting properties of PLCCO samples. (**a**) A typical phase diagram for a hole-doped cuprate La_2−*x*_Sr_*x*_CuO_4_. The AFM, superconducting and spin-glass phases are denoted by AFM, SC and SG, respectively. (**b**) Critical temperatures (*T*_c_'s) determined from the resistivity of PLCCO single crystals annealed by the conventional method reported by Sun *et al*.^26^ (open triangles). (**c**) Schematic description of the protect annealing method. (**d**) The same plot as **a**. (**e**) The same plot as **b** for protect-annealed single crystals reported by Adachi *et al*. (open circles, T. Adachi *et al*., unpublished. *T*_c_ was determined from magnetic susceptibility measurements. (**f**) Magnetic susceptibility of a protect-annealed PLCCO single crystal (*x*=0.10) which shows the *T*_c_ of 27.0 K.

Figure 1 shows the properties of the PLCCO samples. Although PLCCO with *x*=0.10 did not show superconductivity in previous studies^26^ (Fig. 1b), samples protect-annealed as schematically described in Fig. 1c show high *T*_c_ values as shown in Fig. 1e,f (For the superconducting properties, see Supplementary Fig. 1).

**Figure 2:**
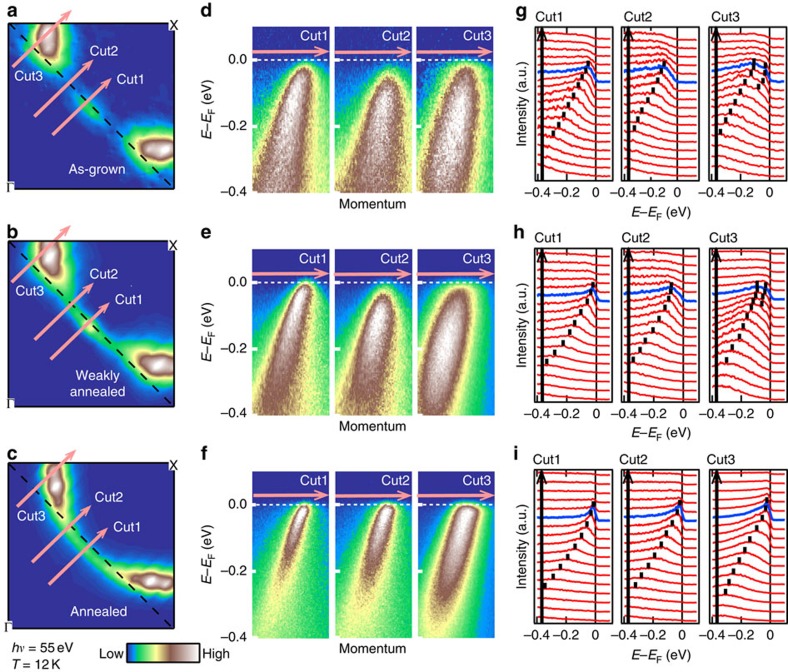
ARPES spectra of PLCCO with and without protect annealing. (**a**–**c**) FS mappings of as-grown, weakly annealed and annealed samples, respectively. The intensity is integrated over ±10 meV of *E*_F_. The suppressed intensities at the hot spots, the crossing points of the FS and the AFM Brillouin zone boundary, are fully recovered in the annealed sample. (**d**–**f**) Intensity plot in energy-momentum space for each sample along each cut indicated in **a**–**c**. (**g**–**i**) EDCs plotted for each cut. Blue EDCs are taken at *k*_F_ positions. Peak positions are marked by vertical bars. The AFM pseudogap that is observed at the hot spots (cut 2) of the as-grown and weakly annealed samples is suppressed in the annealed sample.

In Fig. 2a–c, FS mappings of as-grown, weakly annealed (non-superconducting) and annealed (*T*_c_=27.0 K) samples are shown. In the as-grown sample, the intensities are strongly suppressed around the hot spots due to the AFM order. The intensity partially recovers by the weak annealing, but the FS is still disconnected between the nodal and anti-nodal regions by the presence of the hot spots. This means that the weak annealing was not enough and the influence of AFM correlation still persists. However, in the sufficiently annealed sample, the suppressed intensities at the hot spots were fully recovered, and the entire FS became a continuous circular one. This very simple FS shape is very different from those reported in the previous studies on superconducting samples^5^, in which the intensity is suppressed at the hot spots like the weakly annealed sample reported in the present work. The change induced by the protect annealing is clear also in the band image plots (Fig. 2d–f), and corresponding energy distribution curves (EDCs; Fig. 2g–i) along the cuts through the node, the hot spot and the anti-node for each sample. At the hot spot of the as-grown and weakly annealed samples, the peak is shifted from *E*_F_ towards higher binding energies and at the anti-node the QP peak is split, which can be attributed to AFM correlation. Similar results have also been reported for superconducting samples reduction-annealed by the conventional method^5^, indicating that strong AFM correlation persists even in the superconducting samples. On the other hand, the protect-annealed sample shows that a sharp QP peak disperses towards *E*_F_ without splitting in all the cuts, and the AFM pseudogap is totally absent.

**Figure 3:**
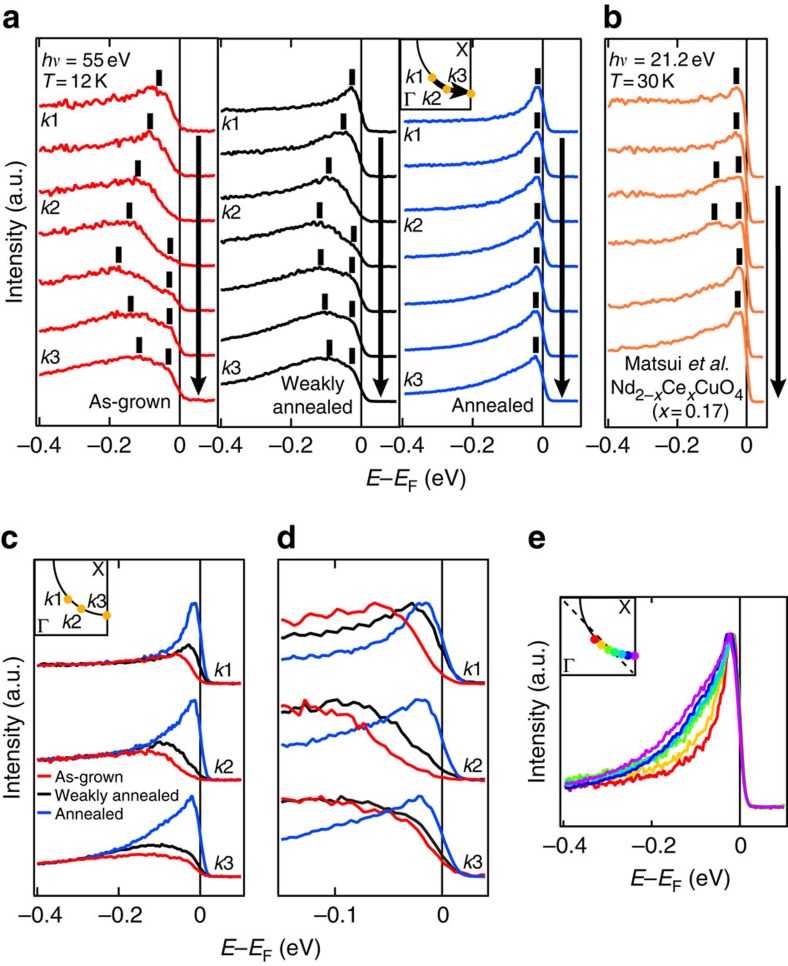
EDCs on the FS of PLCCO. (**a**) EDCs of as-grown (red curves), weakly annealed (black curves) and annealed (blue curves) PLCCO samples along the FS (for *k*_F_ positions, see the inset). Peak positions are denoted by vertical bars. (**b**) The same plot as **a** for overdoped Nd_2−*x*_Ce_*x*_CuO_4_ (*x*=0.17) annealed in a conventional method^19^. (**c**,**d**) Evolution of EDC with protect annealing. EDCs plotted in **c** are normalized to the intensity ∼−0.4 eV. This plot indicates that the spectral weight of the QP peak is dramatically enhanced by annealing. EDCs plotted in **d** are normalized to the peak height. By protect annealing, the gap/pseudogap closes on the entire FS. The momentum positions where the EDCs were taken are indicated in the inset in **c**. (**e**) EDCs on the FS of the annealed sample plotted without offsets. EDCs are normalized to the peak height after the EDC near (*π*, *π*) was subtracted as a background. The inset shows the corresponding momentum positions.

In Fig. 3a, EDCs are plotted along the FS for each sample. The as-grown and weakly annealed samples show a pseudogap between the node and the hot spot, and band splitting between the hot spot and the anti-node. These features are explained by strong AFM correlation as reported in previous ARPES studies^4^,^5^. Surprisingly, all of those features are absent in the annealed sample, and a sharp single QP peak is observed on the entire FS, indicating the suppression of AFM correlation.

The same EDCs are plotted in Fig. 3c,d with different intensity normalizations. According to the plot in Fig. 3c, where EDCs have been normalized to the intensity around −0.4 eV, one can see that the QP peak at *E*_F_ on the entire FS is dramatically enhanced by the annealing. This growth of the QP spectral weight suggests that the scattering of the QPs by impurities or defects is also suppressed by the annealing. Figure 3d, where the EDCs have been normalized to the peak height, shows that the as-grown sample has a gap on the entire FS, consistent with the transport measurements showing an insulating behaviour^25^, and that the gap closes by the annealing, consistent with a previous ARPES measurement reported by Richard *et al*.^17^.

### Quasi-particle scattering rate

**Figure 4:**
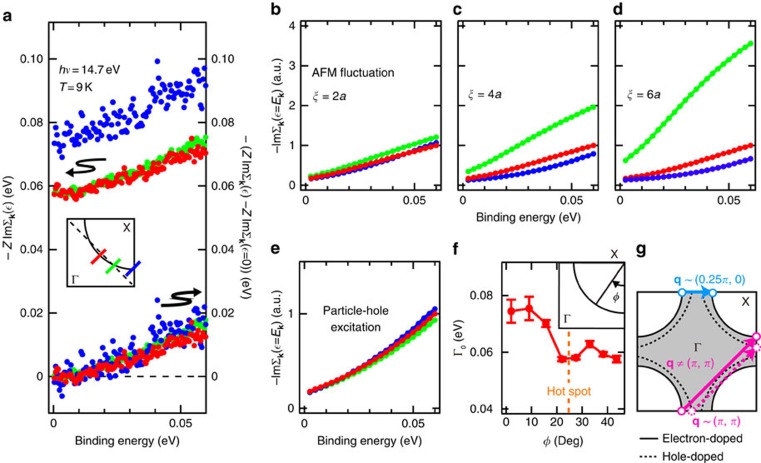
Scattering rate of QPs near *E*_F_ in protect-annealed samples. (**a**) Experimentally obtained scattering rate 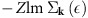 of QPs for the protect-annealed sample with *T*_c_=27.2 K (annealed sample 2) along the cuts indicated in the inset. The dynamical part of the scattering rate 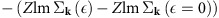 is also plotted at the bottom. (**b**–**d**) Simulation of the dynamical scattering rate 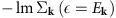 along the cuts indicated in the inset in **a** for AFM fluctuations with the correlation length of (**b**) *ξ*=2*a*, (**c**) 4*a* and (**d**) 6*a*. Calculated 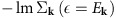 has been normalized to the value at the binding energy of 0.06 eV in the nodal cut. (**e**) The same plot as **b**–**d** for particle–hole excitations. (**f**) Elastic-scattering rate Γ_0_ plotted against the FS angle *φ* defined in the inset. The position of the hot spot is indicated by a dashed vertical line. Vertical bars on the plotted circles represent three times the standard deviations in fitting the data plotted in **a** to a power law function. (**g**) Schematic drawing of elastic scattering of QPs near the anti-node. Solid and dashed curves represent the FSs of electron- and hole-doped cuprates, respectively. Solid and dashed pink arrows correspond to the wave vectors which connects two anti-nodal parts on the FS of the electron- and hole-doped cuprates, respectively. A blue arrow is nesting vector connecting two anti-nodal regions that may lead to charge instabilities in e-HTSCs.

Suppression of the AFM pseudogap around the hot spots enables us to investigate the low-energy physics on the entire FS. The scattering rate of the QPs 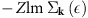, where 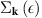 is the self-energy and *Z* is the renormalization factor assumed to be constant in the low-energy region considered here, as a function of QP energy 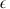, can be evaluated by multiplying the momentum distribution curve width Δ*k* by the Fermi velocity *v*_F_ (Supplementary Note 1). Figure 4a shows thus obtained scattering rate 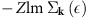 of the annealed sample with *T*_c_=27.2 K (annealed sample 2) along the three cuts, those crossing the node, the hot spot and the anti-node (Cuts 1, 2 and 3 in Fig. 2c). The dynamical (that is, energy-dependent) part of 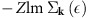 is also plotted at the bottom of Fig. 4a.

We consider two possibilities that the QP created by photoemission is scattered by excitations of electron–hole pairs, which commonly happens in metals, and AFM spin fluctuations. 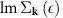 for the particle–hole excitation at low temperatures is approximately given by


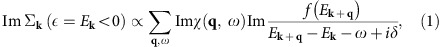


where 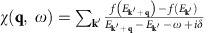 is the Lindhard function^27^,^28^. 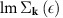 due to AFM spin fluctuations with finite correlation length *ξ* and finite spin fluctuation energy *ω*_SF_ is given by the equation (1) with


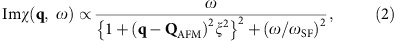


where **Q**_AFM_≡(*π*, *π*)^29^,^30^. Using equation (2) with *ω*_SF_=6 meV deduced from the inelastic neutron scattering measurement of Pr_1−*x*_LaCe_*x*_CuO_4_ (ref. 31), and the experimentally obtained band structure 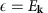 fitted to the tight-binding model^32^ (Supplementary Note 2), 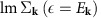 was calculated along the three cuts for different *ξ* values (for the detail of the calculation, see Supplementary Note 1). The calculated 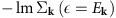 for AFM spin fluctuations and particle–hole excitations are shown in Fig. 4b–d,e, respectively. AFM spin fluctuations with *ξ*≳4*a* (*a*: in-plane lattice constant) yield strong scattering around the hot spot in the low-energy region because low energy AFM spin fluctuations scatter the QPs near one hot spot to another. However, when the correlation length is decreased to *ξ*=2*a*, the scattering at the hot spot is no longer clear as is the case for the scattering by particle–hole excitations. Except for the energy-independent offset (the static part of the QP scattering rate) discussed below, the dynamical part of the QP scattering 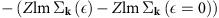, that is, the inelastic scattering of QPs is almost the same among the three cuts as shown in Fig. 4a, suggesting that the dynamical QP scattering in the protect-annealed sample arises from particle–hole excitations as well as from AFM spin fluctuations, if existed, with a short correlation length of *ξ*≲2*a*.

A calculation based on an AFM-phase fluctuation model^9^ has also shown that if the correlation length is as small as *ξ*∼2*a*, an AFM pseudogap becomes invisible as in the spectra of the protect-annealed samples (Figs 2 and 3), while an AFM pseudogap opens for *ξ*≳4*a*. The short AFM correlation length is consistent with *μ*SR measurements on PLCCO single crystals with *x*=0.10 (ref. 33): The rotation of muon spins was observed in as-grown samples, suggesting the existence of long-range AFM order, while in sufficiently protect-annealed samples only fast relaxation was observed, suggesting that AFM correlation is short-ranged. The reduction of the magnitude of the fluctuating spin moment would also contribute to the weakening of the AFM pseudogap. If the latter is the case, the AFM correlation length *ξ* somewhat larger than 2*a* would still be consistent with the ARPES spectra. Thus the absence of AFM correlation signals in the protect-annealed samples indicates that AFM correlation length *ξ* and/or the magnitude of the (fluctuating) local magnetic moments are dramatically reduced by the protect annealing.

As for the static part of the QP scattering rate 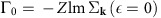, that is, the elastic-scattering rate of QPs, Fig. 4a indicates its enhancement near the anti-node. Note that the elastic scattering is caused by static disorder and should be added to the dynamical scattering, which vanishes at 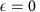. In fact, Fig. 4f shows that Γ_0_ obtained by fitting 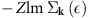 to a power law function 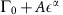 (Supplementary Note 1) increases as one approaches the anti-node. Consistent with these data, the EDC width is also broader around the anti-node as one can see from Fig. 3e, suggesting stronger QP scattering in the anti-nodal region. This tendency has been widely observed in the hole-doped cuprates^34^,^35^,^36^,^37^, suggesting common QP scattering mechanisms both in the hole- and electron-doped cuprates. As for the hole-doped cuprates, coupling with AFM fluctuations peaked at (*π*, *π*)^38^ or scattering between van Hove singularities (for example, between (*π*, 0) and (0, *π*))^39^ have been proposed as a possible origin. In the case of e-HTSCs, however, the (*π*, *π*) scattering mechanism is less effective because the wave vector connecting two anti-nodal parts of the FS are strongly deviated from (*π*, *π*) because of the smaller radius of the FS compared with those of hole-doped cuprates (Fig. 4g). The van Hove singularity scenario is also difficult for e-HTSCs because the singularities lie well (∼400 meV) below *E*_F_ as opposed to ∼100 meV in the hole-doped cuprates. Alternatively, weak nesting between two anti-nodal parts of the FS around (*π*, 0) could enhance elastic scattering of the QPs. If such scattering is strong, incipient charge instability may arise from this FS nesting (Fig. 4g). Recently, charge ordering with **q**∼(0.25*π*, 0) was indeed found both in hole- and electron-doped cuprates^40^,^41^. As for the electron-doped cuprates, the **q** vector is reported to connect either two anti-nodal points or hot spots, and hence it is possible that QPs are scattered between two anti-nodal regions connected by **q**∼(0.25*π*, 0) and the same scattering causes charge instability.

It is interesting to discuss the possible relevance of the present result to the superconductivity with much lower Ce concentration or even without Ce doping reported for thin films and powdered samples of e-HTSCs^21^,^22^,^23^,^24^. In those samples, superconductivity with *T*_c_ as high as the present protect-annealed samples is achieved rather independently of the Ce concentration. Recently, it has been proposed using the local-density approximation combined with dynamical mean-field theory that the AFM long-ranged order is necessary to open a charge-transfer gap in the parent compound of e-HTSCs while Coulomb repulsion without AFM order is sufficient to open the gap in the hole-doped cuprates^42^,^43^. In addition, it has been shown that, when protect-annealed, even extremely underdoped bulk single-crystalline PLCCO (*x*=0.05) becomes superconducting with *T*_c_ values comparable to the present annealed samples (T. Adachi *et al*., unpublished. For the superconducting properties, see Supplementary Fig. 1).

### Carrier concentration

**Figure 5:**
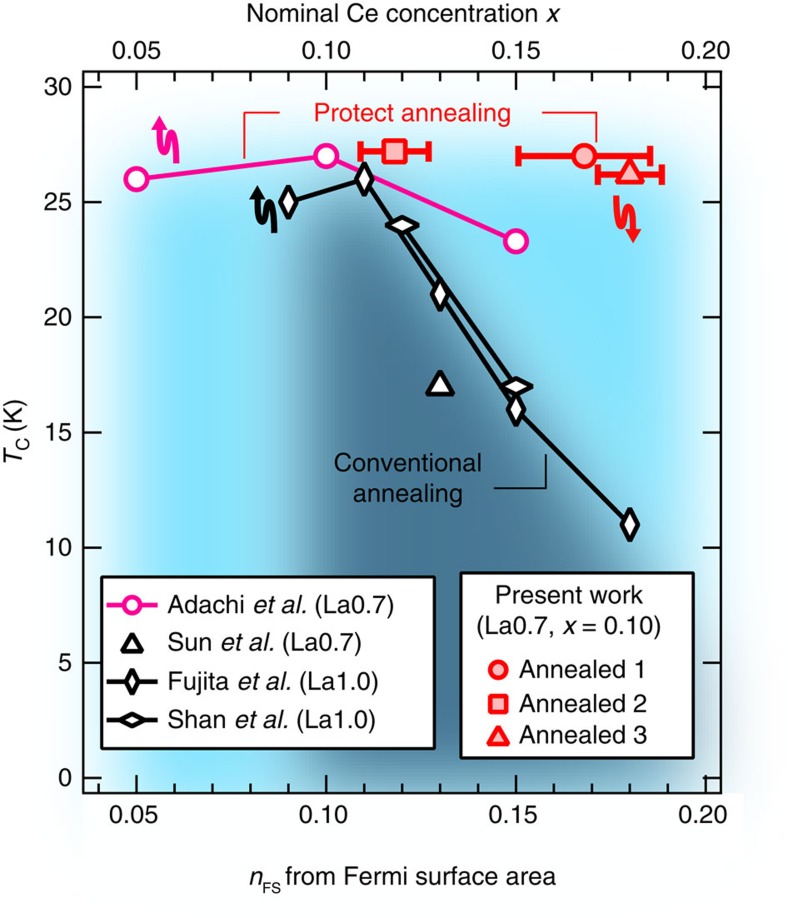
*T*_c_ versus FS area of PLCCO. The values of *T*_c_ of three protect-annealed samples plotted against the doped electron concentration, *n*_FS_, estimated from the area of the FS. Horizontal bars on the plotted data represent three times the standard deviations in fitting FSs to the tight-binding model. For comparison, the values of *T*_c_ of PLCCO and Pr_1−*x*_LaCe_*x*_CuO_4_ single crystals annealed by the conventional method^26^,^31^,^45^, and those of the protect-annealed PLCCO single crystals (T. Adachi *et al*., unpublished.) are also plotted against the Ce concentration *x*. The arrows indicate the axis against which the data with corresponding colour are plotted. The data for PLCCO and Pr_1−*x*_LaCe_*x*_CuO_4_ are denoted by La0.7 and La1.0, respectively. Blue and black shaded regions represent where the superconductivity is realized by protect and conventional annealing, respectively.

The doped electron concentrations of the as-grown and weakly annealed samples estimated from the FS area, *n*_FS_'s, were 0.131 and 0.130, both of which were not far from the nominal Ce concentration *x*=0.10 (Supplementary Fig. 2 and Supplementary Note 2). On the other hand, the *n*_FS_'s of the protect-annealed samples fell in the range from *n*_FS_=0.118 to 0.180, some of which are significantly larger than that expected from the nominal Ce concentration *x*=0.10 (Supplementary Figs 2 and 3, Supplementary Table 1 and Supplementary Note 2). This is distinct from the results of the previous ARPES studies^8^,^17^ in which the FS area was reported to be consistent with Ce concentration. We note that carrier doping by annealing has also been implied by the systematic shift of the Néel temperature by annealing as observed in a neutron scattering study^44^. In Fig. 5, the *T*_c_ values of the protect-annealed samples are plotted against the electron concentration. For comparison, the values of *T*_c_ of PLCCO and Pr_1−*x*_LaCe_*x*_CuO_4_ single crystals annealed by the conventional method^26^,^31^,^45^, and those of the protect-annealed PLCCO single crystals (T. Adachi *et al*., unpublished. For the superconducting properties, see Fig. 1 and Supplementary Fig. 1), are also plotted with respect to the nominal Ce concentration *x*. In the previous studies, the *T*_c_ rapidly decreases with increasing Ce concentration above *x*∼0.11. On the other hand, the present samples maintain high *T*_c_ values compared with all the previous samples up to the highest *n*_FS_ of 0.180 as shown in Fig. 5. This can be understood under the assumptions that Ce doping causes structural disorder and that high *T*_c_ can be maintained if more electrons can be doped without increasing Ce concentration (beyond *x*=0.10).

Although additional electrons are somehow doped in the course of annealing, the suppression of the AFM pseudogap cannot be accounted for only by the electron doping: as shown in Fig. 3b, while overdoped Nd_2−*x*_Ce_*x*_CuO_4_ with *x*=0.17 still clearly shows two peaks in its EDC around the hot spot due to AFM correlation^19^, such a spectral feature is absent in the present ARPES spectra of annealed sample 1 with *n*_FS_=0.168. Furthermore, while weakening of the AFM pseudogap with Ce doping in the previous studies was accompanied by a rapid degradation of superconductivity^19^,^46^, protect-annealed PLCCO (*x*=0.10) shows *T*_c_ as high as ∼27 K despite the absence of the AFM pseudogap, calling for a re-examination of the relationship between superconductivity and antiferromagnetism in the e-HTSCs.

## Discussion

Finally, we discuss possible origins of the excess electron doping determined by ARPES compared with the doped Ce content. In previous studies using inductively coupled plasma (ICP) and X-ray and neutron scattering^15^,^16^, Kang *et al*. detected that 3–3.5% of Cu were deficient in as-grown PLCCO (*x*=0.12) and Pr_1−*x*_LaCe_*x*_CuO_4_ (*x*=0.12) samples and that an impurity phase of rare-earth oxides with ∼1.6% volume fraction was present in annealed samples, concluding that the annealing repaired the Cu defects by creating the Ln_2_O_3_ impurity phase instead of removing apical oxygen. On the other hand, in the present samples, Cu deficiency was not detected before annealing by the ICP analysis within 1% accuracy. Furthermore, the impurity phase of rare-earth oxides was not detected after annealing by X-ray diffraction within the detection limit (∼1%) and by scanning electron microscopy. Another difference from Kang *et al*.^15^,^16^ is that they reported an expansion of the *c*-axis parameter by annealing while we observed a decrease (12.284 and 12.256 Å before and after protect annealing, respectively) as observed in the previous studies by Radaelli *et al*.^11^ and Schultz *et al*.^12^ which supported the removal of apical oxygen by annealing. Taking all these into account, we conclude that the Cu-deficiency scenario^15^,^16^ is not applicable to the present protect-annealed PLCCO samples, and instead oxygen atoms may be removed by annealing from the apical sites^11^,^12^ and/or the regular (CuO_2_-plane and/or LnO-layer) sites^13^ without producing the impurity phase. Therefore, additional electrons should have been introduced by oxygen removal from the regular site, that is, either from the CuO_2_ planes or the LnO layers. Although one cannot further identify the position of the removed oxygen atoms at present, considering the relatively high *T*_c_ of the protect-annealed sample, one can conclude that oxygen removal takes place at atomic sites which induce less disorder than Ce substitution.

In conclusion, we have performed ARPES measurements on protect-annealed PLCCO single crystals with Ce doping of *x*=0.10 with varying annealing conditions. Sufficiently annealed samples showed a *T*_c_ as high as 27.0 K and did not show any signature of AFM fluctuations or the AFM pseudogap, which has been observed in all the other e-HTSCs so far. While the scattering of QPs near *E*_F_ by AFM correlation was not observed at the hot spot in the annealed samples, stronger scattering was observed in the anti-nodal region than in the nodal region, similar to the hole-doped cuprates. This suggests the existence of common scattering mechanisms both in the hole- and electron-doped cuprates although the (*π*, *π*) scattering and the van Hove singularity mechanisms proposed for the hole-doped cuprates do not seem important for the electron-doped cuprates. The protect-annealed samples studied here showed almost the same values of *T*_c_, whereas the actual electron concentration estimated from the FS area varied over a wide range. Thus the intrinsic electronic structure revealed by the present ARPES study will be of great importance to elucidate the mechanism of the high-temperature superconductivity.

## Methods

### Sample preparation

Single crystals of PLCCO with *x*=0.10 were synthesized by the traveling-solvent floating-zone method. The composition of the as-grown crystals was determined to be Pr_1.17_La_0.73_Ce_0.10_Cu_1.00_O_4+*δ*_ by the ICP method by assuming that the content of Pr, La, Ce and Cu amounts to be 3 atoms per f.u. Relative error for each element was ∼±0.01. Three kinds of samples were prepared; as-grown, weakly annealed and annealed samples, among which only the annealed ones showed superconductivity with the *T*_c_ of 27.0 K (annealed sample 1). By protect annealing a bulk polycrystalline PLCCO sample, not shown here, the oxygen content was confirmed to be reduced by 0.03±0.01 by iodometric titration. We also prepared two additional annealed samples with *T*_c_=27.2 and 26.2 K (annealed samples 2 and 3, respectively). The weakly annealed samples were annealed at 650 °C for 24 h, and the annealed samples at 800 ^°^C for 24 h. Bulk superconductivity in the annealed samples was confirmed by specific-heat measurements.

### ARPES measurements

ARPES experiment was performed at beamline 28A of Photon Factory and beamline 9A of Hiroshima Synchrotron Radiation Center (HiSOR). The total energy resolution was set at 28 and 8 meV, respectively. The samples were cleaved *in situ*. The measurements were performed under the pressure better than 2 × 10^−10^ Torr and 5 × 10^−11^ Torr, respectively. Temperature during the measurement was set to 12 K at Photon Factory, and 9 K at HiSOR.

## Additional information

**How to cite this article:** Horio, M. *et al*. Suppression of the antiferromagnetic pseudogap in the electron-doped high-temperature superconductor by protect annealing. *Nat. Commun.* 7:10567 doi: 10.1038/ncomms10567 (2016).

## Supplementary Material

Supplementary Information Supplementary Figures 1-3, Supplementary Table 1, Supplementary Notes 1-2 and Supplementary References
