# Supplementary material for: Suppression of the antiferromagnetic pseudogap in the electron-doped high-temperature superconductor by protect annealing
Source: Nat Commun. 2016 Feb 4;7:10567. doi: 10.1038/ncomms10567 (PMC4743021; doi:10.1038/ncomms10567)
Supplement: Supplementary Information — Supplementary Figures 1-3, Supplementary Table 1, Supplementary Notes 1-2 and Supplementary References [file ncomms10567-s1.pdf]

Supplementary Figures

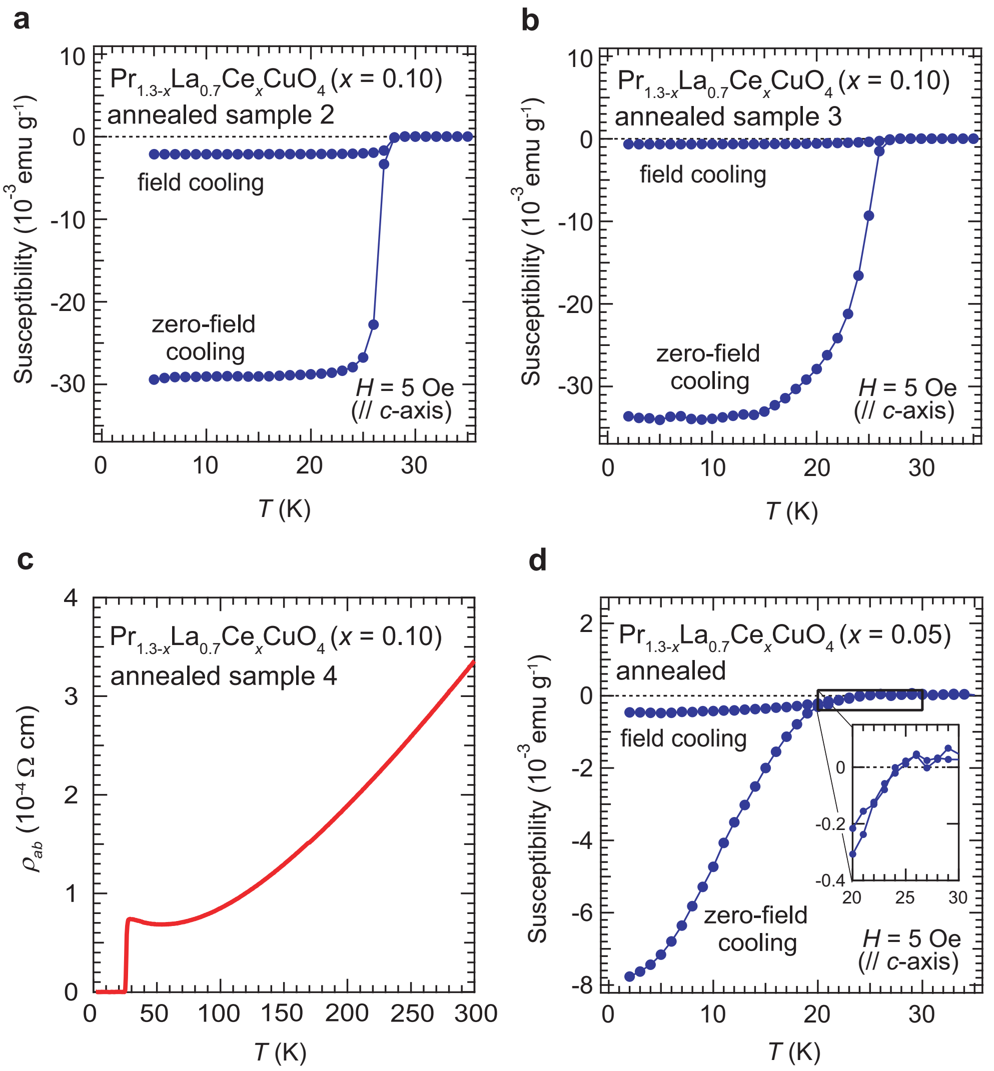

Supplementary Figure 1: **Superconducting properties of the protect-annealed PLCCO samples.** Magnetic susceptibilities of (**a**) $Pr_{1.3-x}La_{0.7}Ce_xCuO_4$ (PLCCO, $x = 0.10$) annealed sample 2 which shows the T_c of 27.2 K and (**b**) PLCCO ($x = 0.10$) annealed sample 3 which shows the T_c of 26.2 K. (**c**) Resistivity of PLCCO ($x = 0.10$) annealed sample 4 which shows the T_c^{zero} of 25.0 K plotted against temperature. (**d**) The same plot as **a** and **b** for annealed PLCCO ($x = 0.05$) which shows the T_c of 26.0 K. The inset shows an enlarged plot of the region near T_c.

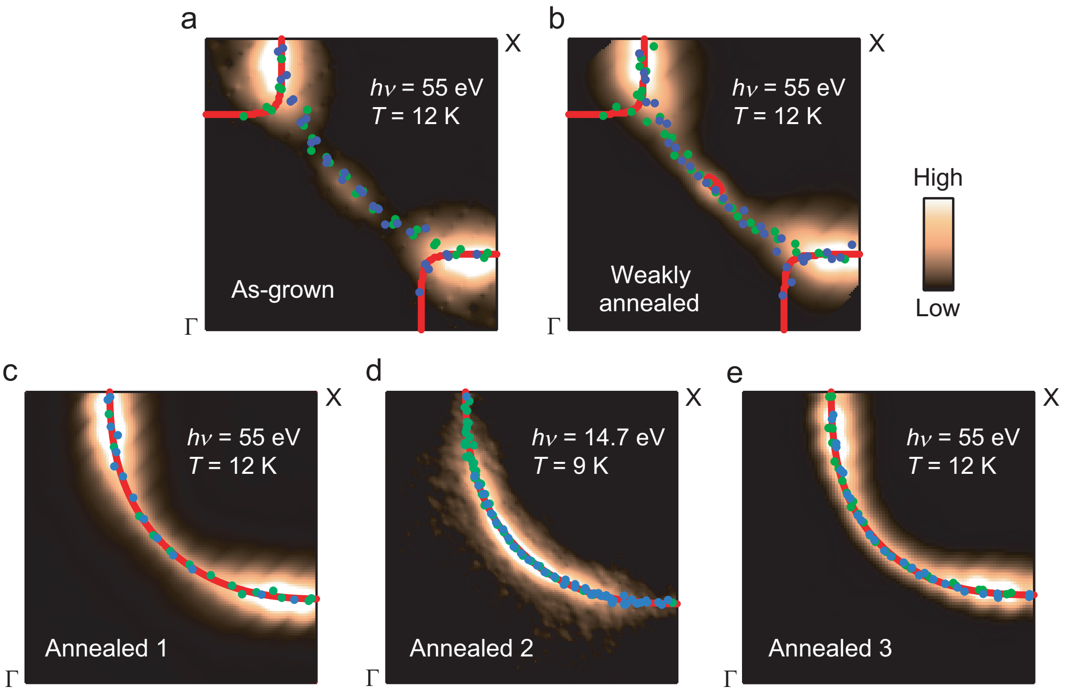

Supplementary Figure 2: **Tight-binding fit of the FS.** (**a, b**) Symmetrized Fermi surface (FS) mappings fitted to the tight-binding model displayed by red curves for the as-grown and weakly annealed PLCCO ($x = 0.10$) samples, respectively. Blue points are the peak positions of the momentum distribution curves (MDCs) at E_F which are obtained in the displayed momentum region or from outside of the displayed region by symmetry operations assuming the four-fold symmetry. Green points have been obtained by symmetrizing blue ones with respect to the Γ-X line. The different distribution of spectral weight in momentum space between annealed samples 1 and 2 is due to matrix-element effects arising from the different photon energies. (**c-e**) The same plots as **a** and **b** for annealed samples 1, 2, and 3, respectively.

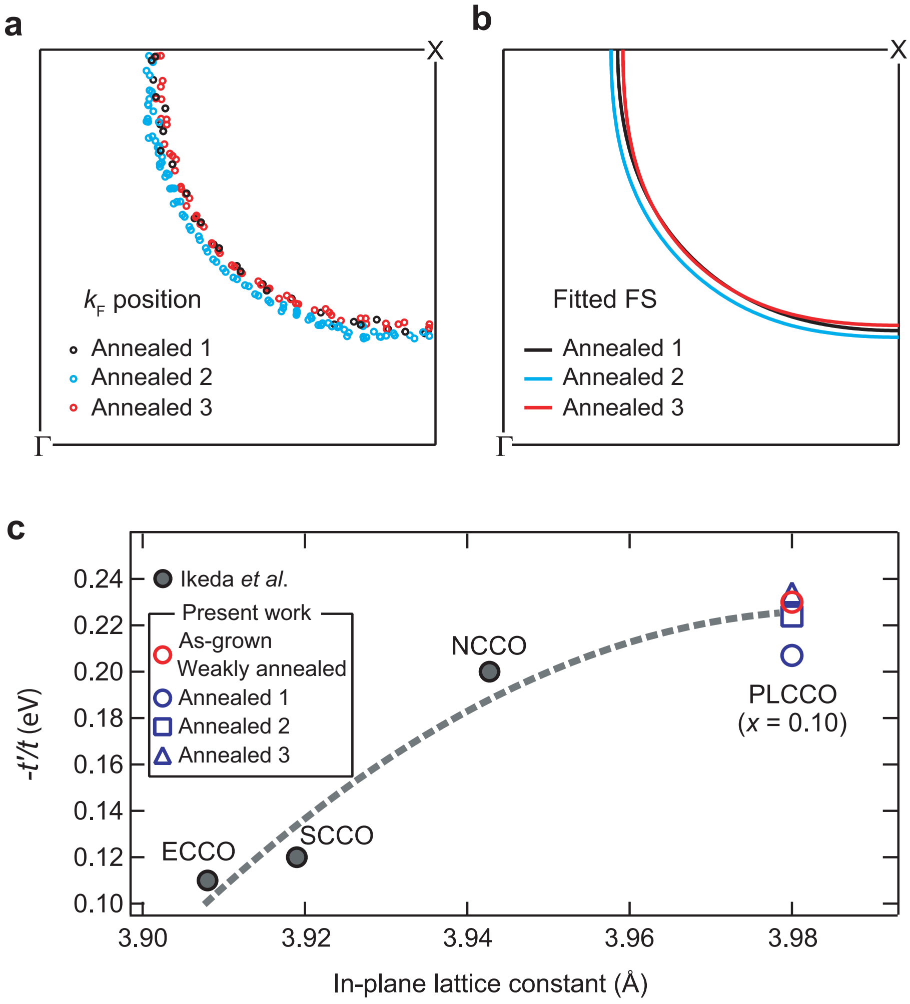

Supplementary Figure 3: **Shape of the FS.** (**a**) k_F positions extracted from Supplementary Figs. 2**c-e** for three annealed samples. (**b**) FSs obtained by fitting k_F positions plotted in **a** to the tight-binding model. (**c**) Relationship between $-t'/t$ and the in-plane lattice constant. The data for $Nd_{1.85}Ce_{0.15}CuO_4$(NCCO), $Sm_{1.85}Ce_{0.15}CuO_4$(SCCO), and $Eu_{1.85}Ce_{0.15}CuO_4$(ECCO) are taken from Ikeda *et al.* [5].

Supplementary Tables

Supplementary Table 1: Parameters obtained by fitting FSs to the tight-binding model. The Fermi surfaces of the three annealed samples were fitted to the model of Supplementary Equation 1 with $\Delta = 0$ and $t''/t' = -0.5$. The error for $-t'/t$ and ϵ_0/t is standard deviation σ of the fit, and that for n_{FS} is $3\sigma_{n_{\mathrm{FS}}}$, in which $\sigma_{n_{\mathrm{FS}}}$ is defined by Supplementary Equation 2.

Sample	T_{c}(K)	$-t'/t$	ϵ_0/t	n_{FS}
annealed 1	27.0	0.207 ± 0.010	-0.102 ± 0.021	0.168 ± 0.018
annealed 2	27.2	0.224 ± 0.005	0.092 ± 0.010	0.118 ± 0.009
annealed 3	26.2	0.234 ± 0.006	-0.133 ± 0.010	0.180 ± 0.008

Supplementary Notes

Supplementary Note 1: Experimental estimates and simulation of the scattering rate of the quasi-particles

The scattering rate of the quasi-particle $-Z\mathrm{Im}\Sigma_{\mathbf{k}}(\epsilon)$, where $\Sigma_{\mathbf{k}}(\epsilon)$ is the self-energy and Z is the renormalization factor assumed to be constant in the low-energy region considered here was evaluated from the ARPES spectra by multiplying the MDC width Δk by the Fermi velocity v_{F}. Δk was estimated by fitting the MDC at each energy to a Lorentzian, and v_{F} was determined by fitting the band dispersion from $E_{\mathrm{F}} - 35$ meV to $E_{\mathrm{F}} - 5$ meV, i.e., above the kink energy [1]. We regarded v_{F} as being constant within this energy range. The Δk and v_{F} values have been corrected for the angle between the cut direction and the FS normal when they are not parallel to each other. Thus obtained v_{F} was 2.1 eV Å, 2.4 eV Å and 1.9 eV Å at the node, the hot spot, and the anti-node, respectively.

To determine the elastic scattering rate Γ_0, $-Z\mathrm{Im}\Sigma_{\mathbf{k}}(\epsilon)$ was fitted to a power law function $\Gamma_0 + A\epsilon^{\alpha}$ in the energy range from E_{F} to 35 meV below it. The error bar was determined by the 3σ of the fitting.

In calculating $-\mathrm{Im}\Sigma_{\mathbf{k}}(\epsilon = E_{\mathbf{k}})$ using equations (1) and (2) of the main text, two-dimensional k space and q space were covered by a mesh of 400×400, ω from 0 eV to 0.1 eV was divided at 5 meV intervals, and δ was set to 0.01 eV. Temperature was set to 9 K, the same condition as the experiment. Calculated $-\mathrm{Im}\Sigma_{\mathbf{k}}(\epsilon = E_{\mathbf{k}})$ has been normalized to the value at the binding energy of 0.06 eV in the nodal cut.

Supplementary Note 2: Tight-binding fit of the FS and band dispersions

The FSs and band dispersions of the as-grown and weakly annealed samples were fitted to the tight-binding model of the square lattice consisting of the Cu $d_{x^2-y^2}$ orbitals with the $\sqrt{2} \times \sqrt{2}$ AFM order as

$$\epsilon - \mu = \epsilon_0 \pm \sqrt{\Delta^2 + 4t^2(\cos k_x a + \cos k_y a)^2} - 4t'\cos k_x a \cos k_y a - 2t"(\cos 2k_x a + \cos 2k_y a), \quad (1)$$

where t, t', and t" are the nearest-neighbor, next-nearest-neighbor, and third-nearest-neighbor transfer integrals, $\pm\Delta$ denotes the staggered potential of the two sublattices (Supplementary Figs. 2a and b). Although this tight-binding model can capture characteristic features of the band structure of e-HTSCs, where AFM correlation is strong, perfect fitting has been difficult because the antiferromagnetism is not a long-ranged one but short-ranged one [2] and the AFM gap Δ is generally **k**-dependent [3], probably due to complicated electron correlation effect that is not considered in the simple AFM tight-binding model. In fact, a variational Monte-Carlo calculation [4] has shown that the AFM gap takes the largest value at $(\pi/2, \pi/2)$, and the smallest value of almost zero at $(\pi, 0)$ and $(0, \pi)$, and hence the dispersion around the band bottom at $(\pi, 0)$ and $(0, \pi)$ can be rather well fitted to the tight-binding model with $\Delta = 0$ [5]. Therefore, for the present as-grown and weakly annealed samples, we determined the value of the parameter t' from the energy position of the band bottom at $(\pi, 0)$ and $(0, \pi)$ using the tight-binding model with $\Delta = 0$.

On the other hand, the FS of the annealed samples can be well fitted to the tight-binding model without Δ, that is, without any signature of AFM correlation, as shown in Supplementary Figs. 2c-e. The different distribution of spectral weight in momentum space between annealed samples 1 and 2 is due to matrix-element effects arising from the different photon energies. The suppression of the intensity at the hot spot could not be detected in all the three annealed samples, and the FSs could be well fitted to the $\Delta = 0$ tight-binding model. Obtained values of the fitting parameters $-t'/t$ and ϵ_0/t are shown in Supplementary Table 1 with their standard deviations σ ($-t"/t'$ has been fixed at 0.5.). The size of the FS varied among different annealed samples as one can see from Supplementary Figs. 3a and b. The doped electron concentrations estimated from the area of the fitted FS, n_{FS}'s, were 0.175, 0.120, and 0.189 for annealed samples 1, 2, and 3, respectively. To estimate the error of n_{FS}, the law of propagation of error,

$$\sigma_{n_{\mathrm{FS}}} = \sqrt{\left(\frac{\partial n_{\mathrm{FS}}}{\partial(-t'/t)}\right)^2 \times \sigma^2_{-t'/t} + \left(\frac{\partial n_{\mathrm{FS}}}{\partial(\epsilon_0/t)}\right)^2 \times \sigma^2_{\epsilon_0/t}}, \quad (2)$$

was used, in which $\frac{\partial n_{\mathrm{FS}}}{\partial(-t'/t)}$ and $\frac{\partial n_{\mathrm{FS}}}{\partial(\epsilon_0/t)}$ were numerically calculated from the change of n_{FS} when $-t'/t$ or ϵ_0/t was changed by 0.0001. As the error bar of n_{FS}, $3\sigma_{n_{FS}}$ was adopted for the safety. The parameter $-t'/t$, which represents the curvature of the FS, are plotted in Supplementary Fig. 3c together with those deduced from other e-HTSCs [5]. The t'/t value of the present PLCCO samples follows the relation ship between t'/t and the in-plane lattice

constant reported by Ikeda *et al.* [5] regardless of the extent of annealing.

Supplementary References

[1] Park, S. R. *et al.* Angle-Resolved Photoemission Spectroscopy of Electron-Doped Cuprate Superconductors: Isotropic Electron-Phonon Coupling. *Phys. Rev. Lett.* **101**, 117006 (2008).

[2] Park, S. R. *et al.* Electronic structure of electron-doped $Sm_{1.86}Ce_{0.14}CuO_4$: Strong pseudogap effects, nodeless gap, and signatures of short-range order. *Phys. Rev. B* **75**, 060501 (2007).

[3] Matsui, H. *et al.* Angle-Resolved Photoemission Spectroscopy of the Antiferromagnetic Superconductor $Nd_{1.87}Ce_{0.13}CuO_4$: Anisotropic Spin-Correlation Gap, Pseudogap, and the Induced Quasiparticle Mass Enhancement. *Phys. Rev. Lett.* **94**, 047005 (2005).

[4] Chou, C.-P. & Lee, T.-K. Variational approach to strong correlation in the photoemission of electron-doped superconductors. *J. Phys. Chem. Solids* **69**, 2944-2948 (2008).

[5] Ikeda, M. *et al.* Effects of chemical pressure on the Fermi surface and band dispersion of the electron-doped high-T_c superconductors. *Phys. Rev. B* **80**, 014510 (2009).
